# Modeling the Effect of Replacing Sugar-Sweetened Beverage Consumption with Water on Energy Intake, HBI Score, and Obesity Prevalence

**DOI:** 10.3390/nu8070395

**Published:** 2016-06-28

**Authors:** Kiyah J. Duffey, Jennifer Poti

**Affiliations:** 1 Department of Human Nutrition, Foods and Exercise, Virginia Tech, 338 Wallace Hall, Blacksburg, VA 24061, USA; 2 Kiyah Duffey Consulting, Inc., 1807 Asher Lane, Blacksburg, VA 24060, USA; 3 Department of Nutrition, University of North Carolina at Chapel Hill, Campus Box #8120, 137 East Franklin Street, Chapel Hill, NC 27516, USA; poti@unc.edu

**Keywords:** sugar-sweetened beverages, water, adults, children, modeling, calories

## Abstract

Sugar-sweetened beverages (SSB) contribute to excessive weight gain through added energy intake. Replacing SSB with water is one strategy that has shown promise in helping lower excessive energy intake. Using nationally representative data from US adults (*n* = 19,718) from NHANES 2007–2012 we examine the impact of replacing SSB with water on Healthy Beverage Index (HBI) scores and obesity prevalence. Replacing an 8-ounce serving of SSB with water lowered the percent of energy from beverages from 17% to 11% (among those consuming 1 serving SSB/day). Reductions in the percent energy from beverages were observed across all SSB consumption groups (1–2 servings/day and >2 servings/day). Among adults there was a 9% to 21% improvement in HBI score when one serving of water replaced one serving of SSB. Using previously published randomized controlled trials (RCT) and meta-analyses of measured weight loss we also predicted a reduction in the prevalence of obesity (observed: 35.2%; predicted 33.5%–34.9%, *p* < 0.05) and increase in the prevalence of normal weight (observed: 29.7%; high weight loss: 31.3%, *p* < 0.05). Our findings provide further epidemiologic evidence that water in the place of SSB can be used as a strategy to limit energy intake and help individuals meet beverage intake recommendations.

## 1. Introduction

Beverages are significant contributors to the daily diets of US adults and have been associated with health through a number of different pathways [1]. Sugar-sweetened beverages (SSB), including soda and sweetened fruit drinks in particular, have remained at the forefront of obesity-related policy debates due to their contribution to excess energy intake and the observation that liquid calories (e.g., beverages) may [2,3,4] or may not [5,6,7] be as satiating as calories in solid form (e.g., food). Regular SSB consumption has been positively associated with increased body weight and risk of obesity [8,9,10,11,12,13], diabetes [9,14,15,16], cardiovascular disease [10], and adverse dietary intake patterns [10,17]. While there is increasingly consistent evidence suggesting that total SSB consumption has been declining over the past 10–15 years [18,19], current consumption levels among many racial/ethnic, age, and income groups nationally (and internationally [20]) remain higher than recommended [19]. For example, while the prevalence of soda consumption among US adolescents decreased between 1999 and 2010, the prevalence of energy drink consumption tripled over that same time period [19].

Maintaining adequate hydration, specifically through water consumption, is associated with a number of beneficial health outcomes including maintaining peak physical and cognitive performance [21], weight management [22] and weight gain prevention [23], and improving metabolic rate [24]. Though there is not a Recommended Daily Allowance for total water intake, the Institute of Medicine’s Food and Nutrition Board suggested a Daily Recommended Intake for adult males and non-pregnant, non-lactating females over 19 years of 3.7 L/day and 2.7 L/day, respectively. Although water intake remains below recommended levels among most segments of the population [24,25], bottled water is poised to overtake soda as the top selling beverage (by volume) [26,27], and powerful new marketing campaigns [28] are hoping to change the way Americans think about water, bottled and tap [29].

Among the top recommendations put forth in the 2015 Dietary Guidelines for Americans is to shift to healthier food and beverage choices [30], specifically that Americans should “shift to reduce added sugar consumption to less than 10 percent of calories per day” [30]. The guidelines additionally state that when choosing beverages “calorie and nutrient contribution to the diet should be considered” and that “calorie-free beverages—especially water—should be favored” [30]. Making individual beverage choice changes can be a challenge because recommendations are not put into the broader context of overall beverage and dietary intake patterns. The Healthy Beverage Index (HBI) [31] was designed to overcome that challenge by providing a system for evaluating overall beverage quality and comparing that intake against standards set forth by the Dietary Guidelines for Americans and the Beverage Guidance Panel [32]. Furthermore, preliminary studies indicate that higher HBI scores are positively associated with cardiovascular health [31,33] and may, therefore, prove a useful tool for evaluating the beneficial impact that changes in beverage consumption patterns might have on health.

The purpose of the present study was to evaluate the potential impact of replacing one serving of SSB with water on HBI score and overweight prevalence.

## 2. Materials and Methods

### 2.1. Study Population

Nationally representative data from US adults (aged 19 and older, *n* = 19,718) from the 2007–2012 National Health and Nutrition Examination Survey (NHANES) and What We Eat in America dietary intake portion of the NHAHES datasets were used in this study. Individuals with missing dietary data (*n* = 3289) were excluded from all analyses leaving a final analytic sample of *n* = 16,429. Sampling methods for continuous NHANES datasets are described in detail elsewhere [34].

### 2.2. Dietary Intake and Beverage Groups

Dietary data were collected using an interviewer administered 24-h recall conducted in-person by trained interviewers during the medical exam in the Mobile Exam Center (MEC) using the United State Department of Agriculture’s (USDA) Automated Multiple Pass Method. A complete description of the survey design is available elsewhere [35,36].

Each food or beverage was recorded by using a discrete food code and matched to nutrient information from the USDA’s Food and Nutrient Database for Dietary Studies (FNDDS) versions 4.1 (2007–2008), 5.0 (2009–2010), and FNDDS 2011–2012 (2011–2012) [37]. Beverages were initially classified according to the Global Food Research Program at University of North Carolina-Chapel Hill Food Grouping System [38], which categorizes beverages separately based on their differential effects on satiety and weight gain. These groups were further condensed in accordance with the Healthy Beverage Index (HBI) guidelines [31] into the following eight groups: Water, Unsweetened Coffee and Tea, Low-Fat (<1.5% and soy) and Skim Milk (hereafter Low-Fat Milk), Diet beverages (including non-calorically sweetened coffee and tea), 100% Fruit Juice, Alcohol (including beer, wine and liquor), Whole Milk (≥1.5% fat), and SSB (including soda, fruit drinks, sweetened coffee and tea, energy drinks and other beverages (e.g., meal replacement drinks).

### 2.3. Healthy Beverage Index

Development and scoring of the HBI has been previously described in detail [31]. Briefly, the ten individual components of the HBI were calculated and scored on a range from 0 to 100 for each participant according to published guidelines. A full description of the final individual 10 components and their scoring criteria can be found in Appendix A. A higher score indicates better adherence to beverage guidelines and an overall healthier beverage intake pattern.

### 2.4. Anthropometrics

Anthropometric data were collected during the in-person medical exam in the MEC by trained health technicians with participants dressed in light clothing using a digital scale and standing stadiometer. Height was measured to the nearest 0.5 cm, and weight was measured to the nearest 0.1 kg. Body Mass Index (BMI) was calculated as weight in kilograms divided by height in meters squared, and then rounded to one decimal place. Individuals were grouped into the following BMI categories: Underweight (<18.5 kg/m^2^), Normal Weight (18.5–24.9 kg/m^2^), Overweight (25.0–29.9 kg/m^2^), and Obese (≥30.0 kg/m^2^).

### 2.5. Statistical Analyses

Analyses were conducted using Stata version 13 (Stata Corp., College Station, TX, USA). Mean (standard error (SE)) baseline levels of beverage and macronutrient intake for children and adults were determined across age groups. The number of 8-ounce (oz) servings of SSB was also determined for all age groups, and individuals were classified as consuming <1 serving, 1–2 servings, or >2 servings over the previous 24 h period. Total energy from all food and beverages and total energy from beverages alone were also calculated and used to generate a measure of percent of energy from beverages. Mean (SE) HBI scores were determined at baseline and recalculated after replacing 1 serving of SSB with 1 serving of water (predicted score). One 8 oz serving was assumed to equal 237 mL of water, and an average reduction of 100 kcal/8 oz SSB was used to estimate changes in energy intake. The change in HBI score was then calculated between the observed score and predicted score, and the percent change in HBI between predicted and observed was then calculated. Student’s *t*-tests, with Bonferroni correction for multiple comparisons, were used to determine statistical significance between observed and predicted HBI scores. Significance level was set at *p* < 0.05.

To predict the impact of replacing one serving of SSB with one serving of water, we applied observed changes in weight from previously published, peer-reviewed randomized controlled trials (RCT) or intervention studies [39], to our population across SSB consumption groups. Specifically, the observed range of changes in weight was from −0.40 kg weight loss to −1.99 kg weight loss [39]. Using these predicted weights, we recalculated BMI and weight classification (normal weight, overweight, obese) to determine the potential impact on overweight and obesity prevalence we might expect from the substitution.

Results were weighted to account for study design and to be nationally representative. The NHANES was approved by the National Center for Health Statistics Research Ethics Review Board. The present study used de-identified publicly available data from NHANES [40] and is therefore exempt from Institutional Review Board approval.

## 3. Results

### 3.1. Sugar Sweetened Beverage Consumption

Differences in intake (ounces (oz)) of individual beverage groups exist across age groups (Table 1). Among adults, water consumption is at its highest for adults aged 19–29 then drops in older age groups. Younger and middle aged adults (aged 19–49) consume the largest absolute number and the largest proportion of their calories from beverages, at 21% (Table 1). The oldest adults (66+) are consuming a greater proportion of water compared to SSBs. 19–29 year olds consume roughly 2.5 times more water than SSB whereas adults 66+ years old consume roughly 6.5 times more water than SSB.

Older adults had the highest proportion of non-consumers and the smallest proportion of high SSB consumers (>2 SSB servings, Table 2). Those considered the highest consumers of SSB (>2 servings/day) were predominately younger and middle-aged adults (aged 19–49). The youngest age group had the largest average SSB consumption among SSB consumers (29.6 oz) and among the age group more broadly (18.5 oz). SSB consumption in the full sample and among consumers decreased among older individuals (Table 2).

Replacing one 8 oz SSB serving with one 8 oz serving of water resulted in considerable change in total energy from beverages and percent of energy from beverages across all age groups (Table 3) with the largest change observed among those who consumed just one SSB serving in the previous 24 h. Among adults who consumed one SSB serving, replacing that with one serving of water resulted in a 33% decrease in total energy and a decrease in the percent of energy from beverages. This resulted in a lowering of their percent of energy from beverages to within the acceptable range [26] of 10%–14% (Table 3).

### 3.2. Impact on Healthy Beverage Index Scores

The distribution of average HBI score among adults is shown in Table 4. Across all age groups, SSB non-consumers had the highest HBI score (indicating greatest adherence to beverage guidelines and recommendations, ranging from 79 to 81 (of 100)). HBI scores were progressively lower with each additional serving of SSB reported as consumed. Older adults (aged 66+) who consumed >2 servings of SSB had the lowest HBI score (mean (SD): 54.4 (0.7)), younger adults (aged 19–29) who were SSB non-consumers had the highest (mean (SD): 81.0 (0.7)).

**Figure 1:**
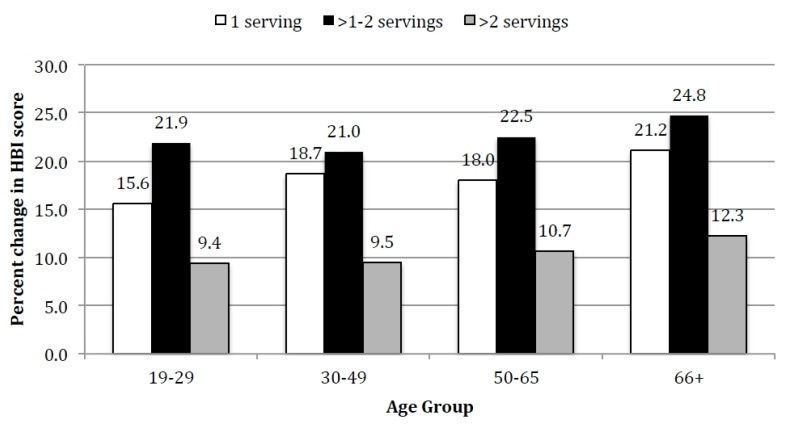
Predicted percent change in HBI score among adults across levels of SSB consumption as a result of replacing one serving of SSB with one serving of water. HBI = Healthy Beverage Index; SSB = Sugar Sweetened Beverages.

Replacing one serving of SSB with water was predicted to improve HBI scores regardless of age groups and across all levels of SSB consumption (Figure 1). At the highest level of SSB consumption (>2 servings SSB), the impact of replacing one serving of SSB with water was greatest for the oldest age group (66+ years, 12.3% increase in HBI score). Regardless of age, replacing one serving of SSB with water had the greatest impact on HBI score among those who consumed between 1 and 2 servings of SSB (range: 21.9%–24.8% increase in HBI score, Figure 1), and this effect was greater for those in older (66+ years) compared to younger (19–29 years) age groups.

### 3.3. Predicted Impact on Overweight and Obesity Prevalence

**Figure 2:**
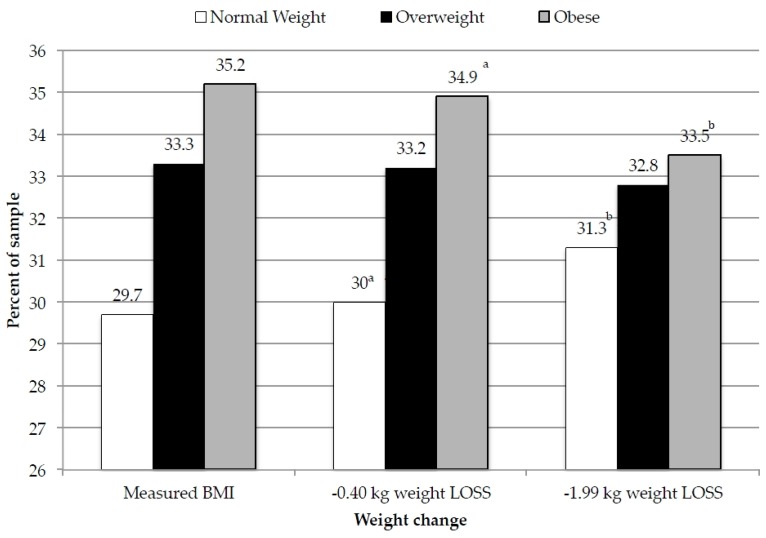
Percent of adults predicted as normal weight, overweight, or obese using predicted weight change [39] associated with substitution of one serving SSB with one serving water. Columns with different letters indicates statistical significance within weight category, *p* < 0.05 with Bonferroni correction for multiple comparisons.

Using weight change values reported in the literature [39], predicted weight change in our sample among those who replaced one serving SSB with one serving of water ranged from −0.40 kg to −1.99 kg among adults (observed over 8 and 6 months intervention follow-up times, respectively). Using these new, predicted, weight values and individual’s measured heights, we estimate a statistically significant reduction in the prevalence of obesity and a statistically significant increase in the prevalence of normal weight BMI among adults who replace one serving of SSB with one serving of water (Figure 2).

## 4. Discussion

This present study confirmed previous reports that a considerable portion of adults are regular consumers of SSB and that those who frequently consume SSB have higher levels of total energy intake as well as energy from beverages. Individuals who consumed a higher number of servings of SSB also had significantly lower HBI scores, indicating that they were further from meeting beverage recommendations compared to those who were SSB non-consumers. In the present study we also report that replacing one serving of SSB with one serving of water significantly improved HBI scores, lowered percent of energy from beverages across intake groups and, in the lowest intake group (1 serving SSB) allowed them to meet the recommendation of consuming <14% of total energy from beverages. Lastly, our findings provide further evidence that water replacement may be an effective strategy for adults concerned about excessive weight. The energy reduction associated with water replacement we predict would significantly lower the proportion of adults classified as obese from 35% down to 32% of the population.

Similar results have been previously reported. A recently published study of more than 18,000 adults using US-based nationally representative dietary intake surveys predicted that a 1 percentage point increase in the proportion of daily plain water was associated with a reduction of roughly 8.5 kcal/day [25]. This finding has been confirmed in experimental and epidemiological studies, where pre-meal water consumption has been documented to reduce meal energy intake in various groups of individuals, including those who are overweight and obese and among older adults [41,42,43]. Given these previously estimated energy savings (68 kcal savings with an 8 ounce replacement of water for SSB [25]), and the fact that roughly 30% of US adults drink one or more servings of SSB/day [44], swapping water for SSB could reduce an estimated 3.9 billion calories from US adult diets daily. Using the observed prevalence of adults consuming at least one SSB serving/day in this study (41.4%), this saving could be as high as 5.8 billion calories saved from American adult diets daily.

Evidence of the impact of SSB reduction on body weight is unequivocal. Using nationally representative data from the UK, Ma et al. [45] report that an estimated 40% reduction in free sugars added to SSB over five years would result in an estimated 1.2 kg body weight reduction and a 1% point decrease in the prevalence of overweight and 2.1% point decrease in the prevalence of obesity among adults [45]. In the US, each one cup per day increment of water intake was inversely associated with weight gain over a 4 year period [23] and a 2013 meta-analysis of RCTs provide fairly convincing evidence that decreasing SSB consumption will decrease the risk of obesity and obesity related diseases such as Type 2 diabetes [8]. Daniels and Popkin additionally state that findings from clinical trials and epidemiologic and intervention studies suggest water has a potentially important role to play in reducing energy intake, and consequently in obesity prevention [46]. These findings have been replicated in non-US settings as well [16,47,48]. However, Hernandez-Cordero et al. [49] found there was no effect of the intervention on triglyceride concentration or on any of the studied outcomes (including weight and waist circumference) in a group of overweight and obese Mexican women [50].

Nonnutritive, or diet, beverages as a replacement for SSB (instead of water) have also been suggested as a means to promote weight loss or encourage weight maintenance, but the findings from these studies are also equivocal. Recently published results from a 1-year RCT evaluating the effects of water compared to low-calorie sweetener (LCS) beverages on body weight in subjects participating in a commercially available weight loss treatment program reported greater 1-year weight loss maintenance among those in the LCS compared to water groups (6.21 kg vs. 2.75 kg respectively) [51]. Others report no difference in weight loss (after 6 months) in the Low-Calorie Beverage compared to Water intervention group [52]. However, they did find that participants in the Water arm of the intervention significantly increased their consumption of fruits and vegetables [53] and had greater reductions in other important markers of health including fasting glucose and improved hydration [53]. Earlier research has shown that dietary and beverage patterns are associated with one another, suggesting that choosing water over diet beverages, as a replacement for SSB, may be associated with other healthy dietary choices [54,55,56].

The studies from which our weight-loss estimates were drawn were based on weight loss observed in randomized controlled trials, which lasted 6 (−0.40 kg weight loss) and 8 months (−1.99 kg weight loss) in a free-living population. In the context of evaluating potential weight loss, this is a meaningful distinction because RCTs that use a controlled feeding methodology may have limited generalizability to a free-living population [57]. Our study did not specifically examine or model the potential compensatory changes in dietary components (including foods and beverages) or in other behaviors like changes in physical activity, except to the extent that these compensatory changes were observed in the free-living population participating in the original weight loss studies themselves [39].

Although trends indicate some decline in intake of sugar sweetened beverages, most Americans are still consuming too many calories from beverages, and from sugar-sweetened beverages specifically. There is ample evidence to suggest that this overconsumption is a contributing factor to excessive weight gain and that options for reducing energy intake, especially the excess energy from beverages, can help limit this excessive weight gain and might even contribute to weight maintenance or weight loss. Results from our study provide further evidence that replacing SSB with water is one potential strategy to achieve these energy reductions and which could benefit adults in the US. Evaluating the impact of water replacement on diet and weight in children and the development of the HBI for a pediatric population are important directions for future research on this topic.

## Figures and Tables

**Table 1:** Reported consumption of beverages, energy, and macronutrients among adults (19+), NHANES 2007–2012.

Beverage Groups (oz)	Age Group			
	19–29	30–49	50–65	66+
Mean (SE), oz				
Water	41 (1.4)	40.8 (0.9)	35 (1.3)	26 (0.7)
Coffee & Tea	5 (0.5)	12.3 (0.5)	20 (0.9)	18 (0.6)
Low Fat Milk	2 (0.2)	2.1 (0.1)	3 (0.2)	3 (0.1)
Diet beverages	3 (0.3)	6.1 (0.4)	7 (0.4)	4 (0.3)
Fruit Juice	3 (0.2)	2.4 (0.2)	2 (0.2)	2 (0.1)
Alcohol				
Wine	0.3 (0.1)	0.6 (0.1)	1 (0.1)	1 (0.1)
Beer	7 (0.6)	6.5 (0.4)	5 (0.7)	2 (0.2)
Liquor	1 (0.1)	0.5 (0.1)	0.4 (0.1)	0.2 (0.03)
Whole Fat Milk	3 (0.2)	2.8 (0.2)	2 (0.2)	3 (0.1)
Sugar-sweetened beverages (SSB)	19 (0.8)	14.1 (0.6)	8 (0.3)	4 (0.2)
Energy (kcal)				
Total kcal from beverages	506 (12)	463 (10)	371 (12)	253 (6)
% kcal from beverages	21 (0.4)	20 (0.03)	18 (0.03)	15 (0.03)
Total kcal	1831 (25)	1820 (19)	1706 (17)	1479 (14)
Nutrients (% of energy)				
Total Fat	32 (0.2)	33 (0.2)	34 (0.2)	34 (0.2)
Saturated Fat	11 (0.1)	11 (0.01)	11 (0.1)	11 (0.01)
Total Sugar	22 (0.2)	21 (0.2)	20 (0.2)	21 (0.1)

**Table 2:** Distribution of SSB consumption and percent consuming among adults (aged 19+), NHANES 2007–2012.

		Mean (SE ^a^) SSB ^b^, oz ^c^		Percent Consumers by SSB Servings, % (SE)			
Age	Sample Size	Full Sample	Among Consumers	Non-Consumers	1	>1–2	>2
19–29	3123	18.5 (0.8)	29.6 (0.9)	37.4 (1.5)	4.3 (0.5)	16.4 (0.9)	41.8 (1.6)
30–49	5411	14.1 (0.6)	27.2 (0.7)	48.3 (1.3)	4.6 (0.4)	14.4 (0.8)	32.7 (1.2)
50–65	4329	7.6 (0.3)	20.8 (0.6)	63.4 (1.2)	4.4 (0.4)	13.3 (0.8)	18.9 (1.1)
66+	3566	4.3 (0.2)	14.7 (0.5)	71.1 (0.9)	7.4 (0.6)	12.1 (0.8)	8.5 (0.5)
19+	16,429	11.7 (0.3)	25.3 (0.5)	53.7 (0.9)	4.9 (0.3)	14.3 (0.5)	27.1 (0.7)

^a^ SE = Standard Error; ^b^ SSB = Sugar-Sweetened Beverages; ^c^ oz = ounces.

**Table 3:** Impact on total energy and percent of energy from beverages resulting from one replacing one serving SSB with one serving of water among adults (aged 19+), NHANES 2007–2012.

	SSB ^a,b^ Consumers, by Servings		
Sample size	**1**	**>1–2**	**>2**
	2544	4181	6081
Total Energy from Beverages			
Reported intake	323 (14)	401 (9)	682 (9)
With water replacement	223 (14)	301 (9)	582 (9)
Percent change	−33.1	−20.2	−14.2
Percent energy from beverages			
Reported intake	17 (0.6)	20 (0.3)	27 (0.4)
With water replacement	11 (0.7)	15 (0.3)	24 (0.4)

^a^ SSB = Sugar-Sweetened Beverages; ^b^ values are % (standard error).

**Table 4:** Distribution of HBI ^a^ score among the full sample and across levels of SSB ^b^ consumption, NHANES adults (aged 19+) 2007–2012.

SSB Consumers, Mean (SE ^c^)					
Age Group	Full Sample	Non-Consumers	1 Serving	>1–2 Servings	>2 Servings
19–29	66.2 (0.6)	81.0 (0.7) ^d^	69.7 (1.5) ^e^	60.3 (0.9) ^f^	54.9 (0.6) ^g^
30–49	68.7 (0.4)	80.0 (0.4) ^d^	68.7 (1.5) ^e^	61.4 (0.5) ^f^	55.2 (0.4) ^g^
50–65	71.3 (0.3)	78.9 (0.3) ^d^	68.2 (1.5) ^e^	60.2 (0.7) ^f^	54.5 (0.5) ^g^
66+	73.5 (0.3)	79.3 (0.4) ^d^	66.7 (1.3) ^e^	58.6 (0.6) ^f^	54.4 (0.7) ^g^

^a^ HBI = Healthy Beverage Index; ^b^ SSB = Sugar Sweetened Beverages; ^c^ SE=Standard Error; ^d,e,f,g^ within rows (age groups), HBI scores with different letters are statistically significantly different from one another and compared to SSB Non-Consumers.

## Abbreviations

The following abbreviations are used in this manuscript:

BMI	Body Mass Index
DOAJ	Directory of Open Access Journals
FNDDS	Food and Nutrient Database for Dietary Studies
HBI	Healthy Beverage Index
MEC	Mobile Exam Center
MDPI	Multidisciplinary Digital Publishing Institute
NHANES	National Health and Nutrition Examination Survey
oz	Ounces
RCT	Randomized Controlled Trial
SSB	sugar-sweetened beverages
USDA	United States Department of Agriculture
